# Advances in Digital Light Processing (DLP) Bioprinting: A Review of Biomaterials and Its Applications, Innovations, Challenges, and Future Perspectives

**DOI:** 10.3390/polym17091287

**Published:** 2025-05-07

**Authors:** Cem Alparslan, Şenol Bayraktar

**Affiliations:** Faculty of Engineering and Architecture, Mechanical Engineering, Recep Tayyip Erdoğan University, Rize 53100, Türkiye; cem.alparslan@erdogan.edu.tr

**Keywords:** DLP, polymer, hydrogel, bioceramics, nanocomposite, smart material

## Abstract

Digital light processing (DLP) technology stands out as a groundbreaking method in the field of biomedical engineering that enables the production of highly precise structures using photopolymerizable materials. Smart materials such as shape memory polymers, hydrogels, and nanocomposites are used as ideal materials for personalized medicine applications thanks to their properties such as superior mechanical strength, biocompatibility, and sensitivity to environmental stimuli in DLP technology. The integration of these materials with DLP enables the production of functional and complex structures, especially in areas such as bone and soft tissue engineering, drug delivery, and biosensor production. However, limited material diversity, scalability problems in production processes, and technical difficulties in optimizing bioprinting parameters are among the main obstacles in this field. This study systematically examines the role of smart biomaterials in DLP-based bioprinting processes. It addresses the innovative applications of these materials in tissue engineering and regenerative medicine. It also comprehensively evaluates its contributions to biomedical applications and discusses future research areas to overcome current limitations.

## 1. Introduction

Advances in modern medicine and biotechnology have accelerated the development of new methods and technologies in line with the goal of improving human health and increasing quality of life [1,2]. In particular, the difficulties in meeting the need for organ transplantation have increased the importance of tissue engineering and bioprinting technologies [3]. In this context, three-dimensional printers (3D printers) and advanced production techniques make it possible to go beyond traditional methods by offering personalized solutions tailored to anatomical and clinical needs [4,5,6,7]. Among these technologies, the DLP method stands out as a production method that offers high precision and resolution. The DLP method enables the production of complex structures through layer-by-layer processing of light-curable (photopolymerized) materials [8,9]. This technology, when combined with bioprinters, becomes an important tool for producing biocompatible and functional organs and tissues. However, the limited properties of the biomaterials used in this process are one of the difficulties encountered in tissue production [10,11]. At this point, it is possible to overcome these difficulties by introducing different materials. In particular, smart materials used in these methods can be sensitive to environmental stimuli (e.g., temperature, pH, humidity), renew themselves, and interact more effectively with biological systems [12,13,14].

**Figure 1:**
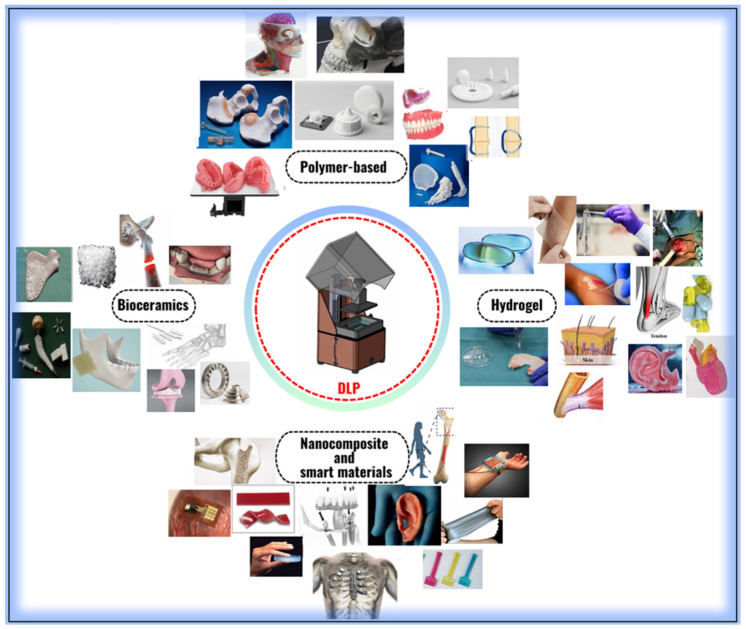
Types of materials used in the production of biomaterials with DLP technology and their application areas.

In recent years, the areas of use of DLP technology in biomaterial production have expanded significantly (Table 1). Various material classes such as polymer-based materials, bioceramics, hydrogels, and nanocomposites offer customized solutions for different medical applications (Figure 1). For example, polymers provide a suitable infrastructure for personalized dental implants and prostheses, while bioceramics play a critical role in bone tissue engineering due to their high mechanical strength and biocompatibility. Hydrogels have great potential in soft tissue applications such as tissue engineering and wound healing. Nanocomposites and smart materials offer superior mechanical and biological properties compared to traditional materials, further accelerating advances in this field. The DLP method enables the high-precision processing of these materials and the production of structures with complex geometries. The potential to provide solutions tailored to individual patient needs, especially in personalized medical applications, makes this technology even more valuable. In this context, the continuous development of methods used in the production of biomaterials not only overcomes current challenges but also forms the basis for new-generation biotechnological applications.

Today, artificial intelligence (AI) stands out as a key enabler in bridging materials science and manufacturing technologies. AI-supported systems optimize manufacturing processes by improving precision and efficiency during material selection and modeling [15]. In particular, machine learning (ML) and deep learning algorithms contribute to the simulation of complex tissue structures and to predicting biomaterial behavior under varying physiological conditions [15,16]. This approach enhances the adaptability of bioprinting systems to clinical requirements, as introduced in the previous section. By analyzing patient-specific biological and genetic data, AI allows for the optimization of biomaterial properties and enables pre-assessment of biocompatibility and functional performance. In doing so, AI accelerates treatment planning and increases the reliability of scaffold design, ultimately contributing to the development of next-generation biomedical applications.

**Table 1:** Materials, techniques, and application areas used in the DLP method.

Material Type	Specific Material	AM Technique	Application Areas
Ceramic Photopolymer resin [17]	ZrO_2_, Al_2_O_3_, SiC, Si_3_N_4_,hydroxyapatite (HA)/Hap, TiO_2_, Yttria-stabilized zirconia, PEGDA poly(ethylene glycol) diacrylate, PETA, HDDA, TMPTA, HEMA, IBOA	Photopolymerization (vat polymerization)	Ceramic prototype manufacturing and industrial components Advanced ceramic material development Precision mechanical parts and durable structures Electronic and biomedical applications
Bioceramics [18,19]	Biphasic calcium phosphate, Hidroksiapatit, β-Trikalsiyum Fosfat, HDDA, TPO, photosensitive resin	DLP	Bone tissue engineering scaffold fabrication Biomedical implants and hard tissue regeneration Fabrication of biocompatible and precise structures
Polymer-based materials [20,21]	Poly L-lactic acid, trimethylolpropane trimethacrylate, poly (N-vinyl carbazole), TMPTMA, NMP	DLP	Bone tissue engineering and regenerative medicine Porous structures with high mechanical strength Biodegradable implants and tissue support elements Skeletal cell culture and in vitro testing
Photopolymer blends [22,23]	Craftsman Beige, Plant-Based Blue, Standard Clear, TMPTA, TEGDMA, DPGDA	DLP	Biomedical applications and personalized structures Precision structures requiring mechanical strength Multi-material systems for testing and prototyping
Bioceramics [24,25]	LithaBone TCP300), hydroxyapatite, ZrO_2_, PEG600DMA, ACMO, ticari slurry (LithaSol 30, Lithoz GmbH), TPO, BAPO, BDMM, ITX)	DLP	Bone tissue engineering scaffold fabrication Biomedical skeletal systems Load-bearing implant structures Regenerative tissue designs with pore optimization
Dental SG^®^ reçinesi/Biyouyumlu reçineler [26,27,28]	Sheraprint SG 100, Dima Print Guide, NextDent C&B MFH (Micro-Filled Hybrid)	DLP/SLA/FDM	Implant guidance and placement accuracy Dental surgical guide manufacturing Low-cost prototypes and guides High-precision dental prototypes
Bioceramics [29,30,31]	Hydroxyapatite, CaTiO_3_, graphene oxide, HDDA, HEMA, TMPTA	DLP	Bone tissue engineering and graft materials Osteoinductive implants and regenerative skeletal structures Load-bearing bone implant support systems Biologically active and durable ceramic structures
PEGDA, DMSO (dimethyl sulfoxide)TPO photoinitiatorEthylene glycol and tolüene [32,33]		DLP	Bone tissue engineering scaffold production Environmental impact assessment and sustainability analyses Life cycle assessment (LCA) study
Bioceramics [31,34,35]	β-TCP (100 nm), hydroxyapatite (HA, 100–200 nm), HDDA, HEMA, TMPTA, Ticari UV-reçine (Anycubic 405 nm)	DLP	Trabecular-like scaffolding for bone tissue engineering Interconnected porous structures that allow nutrient transfer Biomedical implants and regenerative medicine
Polymer-based materials [36,37]	Dimethyl sulfoxide (DMSO), 2-butoxyethyl acetate (EGBEA), and 1:1 DMSO/EGBEA), and degree of PGSA acrylation	DLP	Soft tissue engineering scaffold fabrication Elastic vascular grafts and bioactive structures Biodegradable elastomeric implants Elastomeric scaffolds with optimized mechanical strength
Bioceramics [38,39,40]	β-TCP (LithaBone TCP300), BCP (HA + β-TCP), hydroxyapatite (HA, P100), HDDA, PEGDA-400, urethane acrylate (Neorad U25-20D)	DLP	Scaffold fabrication for bone tissue engineering Osteoinductive implant support systems Bioceramic structures with optimized mechanical strength
PEGDA hydrogel/LAP (lithium phenyl-2,4,6-trimethylbenzoylphosphinate (photoinitiator) [41,42]		DLP	Arthroscopic cartilage repair and tissue engineering Minimally invasive surgical platforms Self-assembling microscaffolds Biomaterials that support cell expansion and regeneration
Photopolymer blends [43,44]	BGB Ultra™, 100–150 nm, Blue Goose Biorefineries, Nano-PCC (CaCO_3_, 40 nm), Nanoclay (Cloisite 20A), hydroxyapatite (HA), calcium phosphate cements, Bioglass^®^, ZrO_2_, fluorcanasite glass ceramics	DLP	Resin stability and flow control High-resolution biomaterial production Reducing microcrack formation
Resin/PLA/VeroWhitePlus™ [45,46]		DLP, FDM, PolyJet	Applications requiring tactile feedback and durability Production of bone models for educational purposes Surgical simulation and planning
Smart materials and hydrogels [47,48]	GelMA, GelGMA, Silk-MA, Silk-GMA, HAMA, HAGM, PEGDA-200/400/700	DLP	Tissue engineering and regenerative medicine Flexible electronic devices and biosensor fabrication Biocompatible scaffolds for cartilage-like tissues Drug delivery and controlled release systems.
Nanocomposite hydrogels [49,50,51]	Polyurethane acrylate (PUA), ZnO nanopartiküller (0.5–2 wt. %), PEGDA, GelMA, barium titanate/hydroxyapatite, DMSO, ethylene glycol, toluene	DLP/SLA/FFF	Targeted drug delivery systems Tissue engineering and cell proliferation support structures Biosensors and flexible electronic sensors Thermal deformable implants and dynamic structures
Smart materials and hydrogels [52,53]	MPA, OPBI (aromatic heterochain polymers), PCL-based LCE with Sm-A liquid crystal moieties	DLP	Brain tissue models and microvascular structures Cell proliferation and homing support structures Biocompatible vessel-like structures Cell adhesion platforms with surface hydrophobicity
ABA triblok kopolimer/Trimetilolpropan [54]		DLP	Biodegradable vessel-like structures and soft tissue implants Production of elastomeric structures for flexible mechanical properties Biomaterial systems with low risk of inflammation
GelMA bioink/LAP [55,56]		DLP	Soft tissue engineering and vessel-like structures Production of fine and complex biostructures requiring support Formation of endothelial-like tissue that supports cell spreading
GelMA bioink/LAP/oxygen-sensitive LiNc-BuO crystals [57,58]		DLP	Oxygen level measurement in bioprinting models Assessment and control of hypoxic microenvironment Long-term oxygen distribution analysis for tissue engineering
Bioceramics/Bioglass [59,60,61]	HA + β-TCP + 45S5 Bioglass^®^ (20 wt%), HDDA + TPGDA/β-TCP + 58S Bioglass (20 wt%), PEGDA + 3,3-dimethacrylate + polypropylene glycol	DLP	Production of porous bioceramic scaffolds for bone tissue engineering Optimization of compressive strength and biocompatibility Surface coating analyses in simulated body fluids Surface designs that increase biological activity through ion Exchange
Acrylamide-based hydrogel/PEGDA and MXene nanosheet [62,63]		DLP	Flexible skin-electronic interfaces and wearable biosensors Ultrasound-assisted adhesion reinforcement mechanism Skin support structures with antibacterial properties Therapeutic patches sensitive to temperature changes
Polymer-based materials [64,65]	DEGDA + PEG + PEG-g-BN, PEGDMA, PCLMA, PU-acrylate	Photopolymerization (vat polymerization)	Biomedical implants and complex geometries Drug delivery systems and shape-shifting structures

The main purpose of this study is to comprehensively examine the research conducted on parts produced by the DLP method and to evaluate the contributions of current research in this field. While the literature often addresses properties such as mechanical durability, cell biocompatibility, and biodegradation in isolation, the combined use of smart materials within DLP-based tissue engineering remains underexplored. Therefore, this review focuses on how DLP technologies, when integrated with smart materials and artificial intelligence, contribute to the fabrication of advanced biomedical constructs. The advantages of DLP in biomedical applications, the functional roles of smart materials, and the supportive role of AI integration are systematically examined. In addition, the study discusses technical and implementation-related challenges, aiming to provide guidance for future research and innovation. By synthesizing existing knowledge and highlighting gaps in the literature, this study contributes to advancing the scientific understanding in the field.

## 2. Technological Fundamentals and Biomedical Applications of DLP

**Figure 2:**
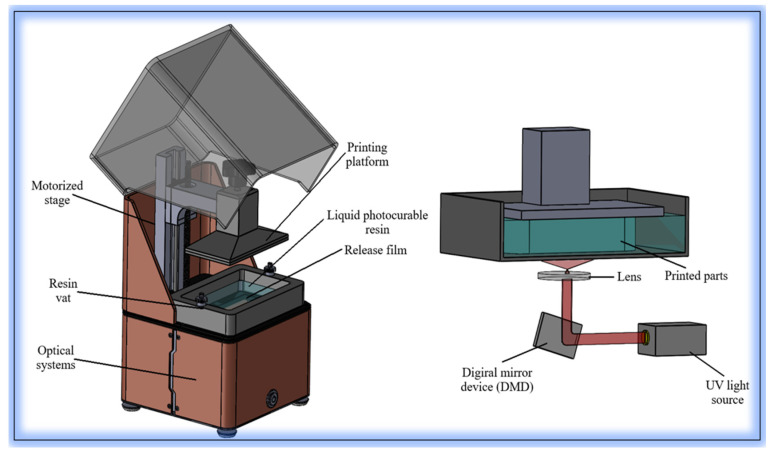
DLP working principle.

The DLP method is a prominent additive manufacturing technique that offers high precision and resolution. It operates by initiating a photopolymerization reaction in a patterned manner through a projected light source, typically within photocurable resins composed of monomers, oligomers, and low-molecular-weight polymers (Figure 2). Unlike conventional 3D printing methods, DLP cures each entire layer simultaneously across the build surface [66]. This approach increases production speed while minimizing material waste. In addition, by using a digital projector instead of a laser, the level of detail is also increased. In DLP systems, the entire image is projected at once using a digital micromirror device (DMD), which contains thousands to millions of individually controlled micromirrors. This allows for precise pixel-level control and sharper feature edges, especially compared to scanning-based methods such as stereolithography (SLA), which rely on tracing the geometry point by point with a laser beam [26,37]. DLP can therefore produce higher-resolution structures more efficiently over larger areas. Additionally, modern DLP systems often incorporate LED arrays and optical homogenizers, which enhance the uniformity of light distribution across the build area and further improve feature consistency and accuracy.

To complement DLP systems, alternative light-based printing methods have emerged. Among them, masked stereolithography (MSLA), also known as LCD-based printing, is widely used. MSLA utilizes an array of light-emitting diodes (LEDs) as a light source, projecting UV light through an LCD screen that acts as a dynamic mask. Each pixel of the screen either blocks or transmits light, enabling layer-by-layer curing of the photopolymer resin. Compared to DLP, MSLA systems generally offer lower resolution due to the fixed pixel grid of the LCD screen, which can lead to pixelation artifacts at the edges of printed features. However, MSLA devices are often more cost-effective and accessible, especially for small-scale laboratory applications. Unlike DLP, where the image is projected through optics and can be distorted near the edges, MSLA provides uniform light distribution over the build area, resulting in more consistent exposure.

In summary, while DLP offers higher optical precision and typically faster cure times per layer due to its projector-based mechanism, MSLA is favored for its affordability, maintenance simplicity, and uniform layer exposure. Both technologies share the fundamental principle of mask-based photopolymerization, but differ significantly in their imaging methods and performance characteristics. In typical DLP-based bioprinting systems, UV or visible light is projected with an intensity ranging from 5 to 20 mW/cm^2^, depending on the device and resin formulation [51,52,53]. The absorption of this radiation is primarily governed by the type and concentration of the photoinitiator (e.g., TPO, LAP), which directly influences the curing depth and reaction kinetics. Under these exposure conditions, localized temperatures can increase by approximately 5–15 °C due to photothermal effects, depending on the resin’s thermal conductivity and absorption coefficient. While DLP is generally considered a low-thermal-load technique, cumulative exposure in large-scale or multilayer printing may cause structural deformation if not properly managed. Therefore, understanding the optical and thermal properties of the resin is critical for process optimization.

In order to more comprehensively reveal the position of DLP technology in the field of three-dimensional bioprinting, a comparative analysis was performed with the extrusion-based, inkjet, and laser-assisted bioprinting techniques commonly used today. The advantages offered by DLP in terms of basic parameters such as resolution, printing speed, cell viability, bioink compatibility, and structural complexity are summarized in Table 2. The table in question provides a holistic perspective between technical competence and biological applicability, allowing the reader to systematically evaluate different bioprinting approaches.

This technology has a wide range of uses in the biomedical field, especially in the production of prostheses, implants, and biological models [67,68]. Compared to traditional manufacturing techniques, the DLP method offers an ideal solution for organ-like structures and models with complex geometries. High resolution, fast production time, and biocompatible material diversity are among the factors that make this method stand out in the field of biomedicine. In particular, detailed structures such as bone grafts, vascular structures, and microstructure elements can be produced more precisely with DLP [69,70,71]. The materials used in the DLP method generally consist of photopolymer resins.

However, in recent years, the use of smart and biocompatible polymers in this process has also become widespread. These materials offer mechanical durability, flexibility, and biological compatibility. DLP-based techniques provide high precision in the fabrication of anatomical models and prosthetic components, making them suitable for surgical planning and biomedical simulations [72,73]. However, limitations exist—light-based curing can lead to deformation in large-scale structures [74,75], and the mechanical strength of photopolymers remains lower than that of native tissues. These deformations are mainly attributed to uneven light intensity distribution across the build platform, which leads to non-uniform polymerization and differential curing shrinkage. For example, projector-based systems may exhibit edge fall-off effects, where the light intensity drops at the periphery of the image. This can result in structural warping, reduced dimensional accuracy, and interlayer delamination, especially in prints exceeding standard build areas. Furthermore, excessive exposure in certain regions may cause localized overheating, contributing to thermal stress-induced deformations. To address this, recent studies have explored the integration of hydrogel-based biomaterials into DLP systems, enabling the production of softer, cell-compatible constructs. Additional advantages such as rapid design iteration, cost-effectiveness, and structural resolution make DLP a versatile tool in clinical workflows (Table 3). These features have notably contributed to simulation-based preoperative planning and device prototyping, supporting advances in translational biomedical applications [76].

As a result, DLP technology has introduced transformative capabilities in biomedicine and tissue engineering by enabling the rapid fabrication of complex, cell-compatible microstructures with micrometer-level resolution. Its ability to produce patient-specific scaffolds, vascularized networks, and bioactive implants within a short timeframe makes it particularly suited for personalized medicine, regenerative therapies, and surgical planning applications. Ongoing advancements in smart material development and process optimization are expected to further extend DLP’s applicability in more complex clinical scenarios, including functional organ fabrication. Therefore, research on DLP systems remains a vital component in the evolution of next-generation bioprinting platforms. In the following sections, selected case studies are presented to demonstrate how DLP-based strategies have been effectively implemented in scaffold fabrication, particularly within the context of bone tissue engineering.

### 2.1. Case Studies on the Development of Porous Scaffolds for Bone Tissue Engineering

Kordi et al. developed poly-L-lactic acid (PLLA)-based scaffolds reinforced with graphene oxide (GO) nanoparticles using a DLP-based photopolymerization approach for bone tissue engineering. In this study, a photocurable PLLA-based resin formulation was synthesized by combining low molecular weight PLLA oligomers with a reactive diluent (NMP), a trifunctional crosslinker (TMPTA), and a photoinitiator (TPO). This mixture enabled light-induced polymerization, allowing DLP-based fabrication despite PLLA’s inherent thermoplastic nature. GO was incorporated into the resin at different weight ratios (0.5%, 1%, and 1.5%) to enhance mechanical and degradation performance. The scaffolds were designed with a nominal pore size of 750 µm and 40% porosity, and heat treatment was applied at 85 °C for durations of 0, 6, 12, and 18 h. Mechanical tests revealed that increasing both GO content and heat treatment duration significantly enhanced compressive strength, with improvements ranging from 119% (0.5 wt.% GO, 6 h) to 235% (1.5 wt.% GO, 18 h), relative to untreated PLLA scaffolds. Post-treatment pore sizes ranged from 500 to 750 µm, and the overall porosity decreased slightly, remaining within the range of 30–40%. The highest compressive strength of 48.6 MPa was achieved in samples with 1.5% GO after 18 h of heat treatment. The incorporation of GO also improved surface hydrophilicity by reducing the water contact angle by 18%. Degradation studies in phosphate-buffered saline over 8 weeks showed up to 40% weight loss in 1.5% GO-containing scaffolds. The untreated samples exhibited faster degradation due to microcrack formation. MTT assays confirmed that GO incorporation had no detrimental effect on cell viability, with 1.5% GO samples maintaining over 70% viability. All groups, except 1.5% GO, showed higher optical density values than the DMEAM control group on day 7. These PLLA/GO scaffolds, fabricated via a DLP-compatible resin formulation, exhibit strong potential for applications in bone grafts and dental implants due to their enhanced mechanical performance, controlled degradability, and cytocompatibility [80]. Bahati et al. investigated vat photopolymerization (VPP) technologies for bone tissue engineering applications. In this study, the mechanical strength, biocompatibility, and production cost of skeletal structures produced with DLP and SLA techniques were discussed. It was stated that SLA and DLP are promising for the production of biomaterials with complex porous structures at high resolution and low cost. In DLP and SLA technologies, photopolymer resins are hardened as a result of the curing process with light energy. While DLP projects light on the entire layer at once, SLA solidifies the layers one by one using a single laser point. Micrometer-level resolution was achieved in both methods. The materials used were selected from biocompatible and biodegradable photopolymer resins. In mechanical tests, elastic modulus, compressive strength, and crack formation rate were evaluated. Hydroxyapatite (HA) ceramic scaffolds produced with DLP exhibited compressive strength of 5.6–18.4 MPa and elastic modulus of 2.4–5.9 GPa. In the samples produced with SLA, although precise geometry was obtained, the process was slower. The pore sizes of the produced scaffolds were measured up to 500 µm, and the porosity ratio varied between 40 and 90%. Higher porosity supported cell proliferation; however, a decrease in mechanical strength was observed. In vitro studies conducted on structures produced with SLA and DLP yielded successful results in terms of cell adhesion, viability, and proliferation. Vascularization and bone formation were observed, especially in 3-month in vivo tests. In calcium phosphate-based scaffolds produced with SLA and DLP methods, different mechanical values were obtained depending on the chemical composition and sintering temperature. For example, hydroxyapatite-zirconia composites prepared using DLP have yielded results close to natural trabecular bone, with a tensile strength of 15.4 MPa. DLP and SLA are techniques with high potential for the production of personalized implants. While SLA offers high resolution in complex geometries, DLP provides faster production. In the future, it is expected that these techniques will be used more widely in the medical field with the development of multi-colored resins and cell-friendly bioresorbable materials [81].

### 2.2. Case Studies on the Utilization of Natural Reinforcements and Advanced Biomaterials

Chivate and Zhou extensively studied the fabrication of micropatterned surfaces using additive manufacturing (AM) techniques. The functional properties of microstructures found in the natural world provide inspiration for biomimetic approaches in engineering. Micropattern technologies have a wide range of applications, especially in areas such as surface hydrophilicity, adhesion control, and light management. The study divided microstructure fabrication techniques into two main categories: direct and indirect methods. Direct methods create microstructures on the surface without using molds, while indirect methods use negative molds or lithographic processes. Major techniques include inkjet printing, DLP, and microlithography. The DLP method allows rapid fabrication of high-resolution layered structures with large-area photopolymerization. Micro-SLA methods are widely used, especially in the fabrication of complex geometries, such as microlenses and microneedle arrays. In order to enhance the light collection capacity in optical devices, microlens arrays fabricated via DLP have demonstrated high geometric accuracy and cost-effectiveness compared to conventional microlithography techniques [82]. These arrays are particularly useful in imaging systems, light sensors, and optical communication devices due to their rapid prototyping and fine resolution. Micropattern designs inspired by the Namib beetle and shark skin have enabled superhydrophobic and water-harvesting properties on surfaces fabricated using DLP and inkjet techniques. Compared to the smooth-surfaced controls, the water retention capacity of these structures increased by approximately 20%, as reported by Chivate and Zhou. The structural stability of microdroplet arrays produced, especially with inkjet printing, has been optimized according to droplet size and contact angle. High-precision conductive paths have been obtained in electronic circuits. AM technologies, particularly DLP and inkjet methods, have enabled the fabrication of high-resolution microscale structures such as microneedles, microlenses, and bioinspired droplet arrays, which are essential in applications ranging from targeted drug delivery to optical imaging. More complex and precise surfaces can be obtained thanks to DLP, SLA, and inkjet methods. The use of microstructures in biomedical applications offers great potential for personalized medical devices and advanced optical systems in the future [82]. Wang et al. developed completely natural ceramic porous bone scaffolds using natural calcium phosphate ceramic whitlockite (WH) and natural silicate ceramic wollastonite fiber (WF) using DLP additive manufacturing technology. In the study, the mechanical strength, biodegradation behavior, biomineralization capacity, and cell response of the scaffolds were comprehensively investigated. This study presents promising results for personalized bone implants. Whitlockite/Wollastonite scaffolds were fabricated on a desktop DLP device using ceramic slurry with a solid loading ratio of 75%. Sintering processes were carried out at temperatures of 900 °C, 1000 °C, and 1100 °C. Samples with different WF ratios (0%, 5%, 10%, 15%, 30%) were prepared, and mechanical tests, biodegradation experiments, biomineralization studies, and cell culture tests were performed. WF addition increased the compressive strength of the scaffolds from 20 MPa to 31 MPa. Samples containing 10% WF showed maximum strength (31.0 MPa, elastic modulus: 757 MPa). However, when the WF ratio was increased to 30%, the mechanical properties decreased due to fiber accumulation. Increasing the sintering temperature reduced porosity and increased density by creating stronger bonds between ceramic particles. Samples with a 50% design porosity exhibited suitable properties for optimum cell growth and mechanical support. In the degradation tests conducted in Tris-HCl solution, the degradation rate increased with WF addition. Especially in scaffolds containing 30% WF, the pH value increased to 8.3. The release of magnesium and silicon ions showed effects supporting osteogenesis processes. Ca-P minerals were formed in scaffolds kept in simulated body fluid (SBF) solution for 2 weeks. The amount of mineral deposited on the surface increased with WF content, and the surface was completely covered in scaffolds containing 30% WF. In culture tests conducted with MC3T3 osteoblast cells, it was observed that WF supplementation promoted cell proliferation and differentiation. However, a decrease in cellP number was detected on the seventh day due to the increase in pH in groups containing 30% WF. Whitlockite ceramic bone scaffolds with WF supplementation exhibited improved performance in terms of mechanical strength and biological activity. A 10% WF ratio was determined as the formulation providing the highest mechanical strength and optimum biological compatibility. The study demonstrated the potential of using natural ceramic materials in personalized bone implants with additive manufacturing methods [83].

Elsayed et al. investigated the manufacturability of highly porous scaffold structures using Biosilicate^®^ glass-ceramic powders in direct ink writing (DIW) and DLP techniques and additive manufacturing methods. Biosilicate^®^ stands out with its biocompatibility, osteoinduction, and antibacterial properties and has been presented as an alternative material to 45S5 Bioglass^®^. Biosilicate^®^ powders (particle size < 5 µm) were used in DIW and DLP systems by preparing ink formulations and photosensitive resin mixtures. Samples were sintered between 1000 °C and 1100 °C, and their microstructures were examined using an optical microscope and a scanning electron microscope (SEM). Mechanical strength was measured with a universal testing machine, and X-ray diffraction (XRD) analyses were used to determine crystalline phases. The skeletons produced with the DIW method showed a compressive strength of 6 MPa, with a volumetric porosity of 52 ± 2%. In the samples produced with the DLP method, a strength of 0.9 MPa was measured, with a porosity of 78%. The DLP skeletons sintered at 1100 °C showed a strength of 3.2 MPa with the increase in the crystallization level. However, due to the high porosity, micro-voids limited the mechanical properties. After sintering, sodium-calcium silicate (Na_2_CaSi_2_O_6_) was dominant among the crystal phases, and the surface roughness showed suitable properties for cell adhesion. While DLP allowed the production of more complex geometries, the DIW method produced more durable skeletons with a higher solid content. Filament fusion at the bonding points of the DIW skeletons provided homogeneity. Biosilicate^®^ glass-ceramic is a promising candidate for biomaterials with osteogenic properties. The DIW method offers high mechanical strength for biomedical applications, while the DLP method is advantageous in terms of aesthetics and geometric complexity. In the future, the aim is to increase mechanical performance by optimizing the microstructure of pore walls [84]. Bucciarelli et al. investigated the fabrication of shape memory structures using methacrylated silk fibroin (Sil-MA) by additive manufacturing. Sil-MA is crosslinkable under UV light and enables 2D and 3D personalized implant designs for bone tissue engineering. In the study, the mechanical properties, biocompatibility, and osteogenic potential of silk fibroin hydrogels were evaluated. Sil-MA hydrogel structures were produced using DLP and pneumatic extrusion techniques. In total, 10–20% Sil-MA and 0.1% LAP were used as photoinitiators in the recipes. Samples with crosslinking times ranging from 5 to 30 s were prepared and chemical changes were confirmed using FTIR and NMR analyses. Cell biocompatibility tests were performed with MRC5 human lung fibroblast cells and human adipose stem cells (ADSC) according to the ISO 10993 protocol [85]. The increase in crosslinking time increased the hydrogel stiffness. The swelling ratio of samples with 30 s of UV exposure was limited to 45%, while the swelling ratio reached 90% in samples with 5 s of exposure. In the case of insufficient crosslinking, the elastic modulus of the hydrogel structures decreased. The Sil-MA structures were able to return to their original shape after rehydration after being folded through a syringe tip. The LDH test showed that Sil-MA films were not cytotoxic. According to the Pico Green test, cell proliferation increased by 300% on day 6. Alizarin Red staining confirmed increased mineralization on ADSC cells. While the DLP method provided higher resolution and detail, the pneumatic extrusion method caused the filament diameter to expand, but allowed for thicker 10-layer structures. Methacrylated silk fibroin-based hydrogel scaffolds are a promising candidate for applications such as filling bone voids due to their osteogenic potential and shape memory properties. In the future, it is recommended to verify the effectiveness of these structures in bone regeneration using in vivo tests [86].

### 2.3. Case Studies on Hydrogel Systems and Smart Materials via DLP Printing

In a study conducted by Ding et al., a functional double network structured hydrogel system (Z/P/CO) reinforced with zinc-curcumin nanoparticles (ZnCur NPs) and short peptide RL-QN15 was developed for the treatment of infected diabetic wounds. The hydrogel matrix was formed by both Schiff base and photochemical crosslinking methods using modified hyaluronic acid (OHAMA) and chitosan derivatives (CSA). ZnCur NPs stand out with their antioxidant, anti-inflammatory, and antibacterial properties, while RL-QN15 peptide supports angiogenesis and cell proliferation. In in vitro tests, it was reported that Z/P/CO hydrogel showed high biocompatibility and proliferation ability on HDF cells, and provided immune modulation by promoting the transition from the M1 to M2 phenotype in RAW264.7 macrophages. In addition, it has been shown that the hydrogel adheres well to the wound, provides a moist healing environment, and provides long-term antibacterial activity due to its self-healing and tissue-adhesive properties. In the experiments conducted on the in vivo diabetic wound model, almost complete wound closure, reduced inflammation, increased collagen deposition, and vascularization were observed in the Z/P/CO group by day 17. These findings indicate that the Z/P/CO hydrogel platform is a promising multifunctional strategy for accelerating infected diabetic wound healing [87]. Ding et al. developed methacrylated hyaluronic acid (HAMA)-based hydrogel-formed microneedle systems (V-R-MNs) containing VEGF and Ritlecitinib, supported by poly(hydroxyalkanoate) (PHA) nanoparticles for the treatment of androgenetic alopecia (AGA). While HAMA was used in the production of microneedle tips with its photopolymerizable biocompatible structure, hydrophobic Ritlecitinib was encapsulated in slowly degradable PHA nanoparticles, and long-term release was provided. In in vitro tests, it was reported that these structures had high skin permeability and low cytotoxicity and showed high viability, cell migration, and angiogenesis effects in HDF and HUVEC cells. In the in vivo AGA mouse model tests, the V-R-MN group showed significant increases in melanin accumulation, hair follicle development, vascularization, and Ki67 proliferation marker levels, and significant decreases in IL-6 and TNF-α expression levels were also detected. A faster transition to the anagen phase and wider hair coverage area were obtained compared to minoxidil and topical Ritlecitinib applications. These results show that dual drug carrier hydrogel microneedle systems combined with mechanical stimulation offer a non-invasive, effective, and long-lasting treatment strategy in the treatment of AGA [88]. In this context, innovative solutions offered by smart materials in optimizing the performance of biomedical structures produced with the DLP method open new horizons in biomedical engineering and personalized medicine applications. Smart materials offer significant advantages where traditional biomaterials are inadequate thanks to their features such as sensitivity to environmental stimuli, mechanical strength, shape memory, self-healing ability, and superior compatibility with biological systems. Recent studies have also emphasized the role of natural biopolymers such as silk fibroin, gelatin, and alginate—particularly in combination with photo-crosslinkable agents like PEGDA—in developing functional bioinks for vat photopolymerization systems [89]. The use of these materials, especially in advanced applications such as tissue regeneration, implant design, and biosensor production, expands the boundaries of bioprinting technologies and enables the production of more functional and long-lasting biomedical products. In this direction, the multidimensional role of smart materials in the DLP method will be evaluated comprehensively in terms of critical parameters such as biocompatibility, biodegradation behavior, mechanical strength, and cell interaction, and their contributions to biomedical applications will be discussed in detail.

### 2.4. Case Studies on Direct Bioprinting with Cell-Containing Bioinks Using DLP

In recent years, the field of application of DLP technology has expanded to include direct bioprinting processes with cell-containing bioinks, and this stands out as a significant advance in the fields of tissue engineering and regenerative medicine. Various studies have shown that functional and complex biostructures are successfully produced by encapsulating living cells in DLP-printable hydrogel structures. For example, with the low-temperature DLP system developed by Yang and colleagues, a photopolymerization process was successfully carried out at 25 °C using low-concentration collagen methacrylate (ColMA) bioinks, which preserved the bioactivity of collagen that is typically compromised at higher temperatures. This approach enabled the fabrication of scaffolds that supported fibroblast adhesion, proliferation, and differentiation while minimizing thermal denaturation and enzymatic degradation [90]. Kumar and his team developed GelMA bioinks optimized for visible light-operated DLP systems and showed that astrocyte and fibroblast cells could grow within the structure with viability rates exceeding 90%, as confirmed by the live/dead assay and long-term culture analysis [91]. Zhang and his colleagues discussed the usability of light-cured bioinks in tissue engineering, emphasizing that many tissue models such as heart, bone, and vein can be produced with DLP [92]. In addition, Lee and his team reported that photopolymerization-based 3D printing systems provide successful results in the printing of microvascular networks that promote angiogenesis, especially in hypoxia-related diseases [93]. In the latest review by Fan et al., advanced bioengineering examples using cell- and growth factor-enriched DLP platforms for vascularized bone regeneration are presented [94]. Finally, Fang et al. demonstrated successful regeneration examples in multiple tissue types with parametric strategies for customizing DLP systems, from the selection of cell viability-sensitive photobondable bioinks to light dose adjustment [95]. These studies demonstrate that DLP bioprinting technology has increasing potential for the production of biofunctional tissues by simultaneously printing biomaterials and living cells, providing a strong foundation for future developments in personalized medicine.

Table 4, prepared within the scope of this study, summarizes the direct cell-containing bioprinting applications of the DLP method in the recent literature. In these examples, where different bioinks (e.g., GelMA, PEGDMA, CSMA) were combined with various cell types (fibroblasts, mesenchymal stem cells, endothelial cells, etc.), over 90% viability rates, long-term culture capability, and high structural resolution were achieved. In addition, the light sources, wavelengths, and application areas (e.g., bone, skin, vascular, and cartilage regeneration) used in each study are specified. This table clearly demonstrates the diversity and potential of DLP technology for cell-supported structure production in tissue engineering.

**Table 4:** Selected cell-laden DLP bioprinting studies.

Study	Bioink	Cell Type	Cell Viability (%)	Light Source	Application
Su et al. (2021) [96]	GelMA + Si-HAp	MG63, hBMSCs	>90%	Visible light (maskless DLP)	Bone regeneration
Zhu et al. (2017) [97]	GelMA	Endothelial + support cells	High (qualitative)	mCOB (optical bioprinting)	Pre-vascularized tissue fabrication
Duong & Lin (2023) [98]	GelNB + PEG4SH + QK peptide	HUVEC	>90%	DLP (visible light)	Vascularized scaffold printing
Choi et al. (2023) [99]	Gel-GMA + Silk-GMA	Keratinocyte, fibroblast, endothelial	High (after 4-week culture)	DLP (405 nm LED)	Artificial skin and wound healing
He et al. (2024) [100]	CSMA + CoLMA	Keratinocyte, fibroblast	Good proliferation	DLP (blue light)	Biomimetic skin engineering
Tilton et al. (2023) [101]	GelMA + PEGDA	Human cells (unspecified)	>72% (post-injection)	Visible light (DLP)	Injectable regenerative scaffold
Xie et al. (2022) [102]	GelMA + CAM microtissue	Chondrocytes (from microtia)	92–98% (over 20 days)	DLP (14 mW/cm^2^, 405 nm)	Auricular cartilage regeneration
Chang et al. (2022) [103]	GelMA + PEGDMA	Human fibroblasts	High (after 5-day culture)	DLP (25 µm layers, 5–8 s/layer)	Microchannel-based soft tissue engineering

## 3. The Role of the DLP Method in the Production of Smart Materials

Smart materials have revolutionized biomedical applications due to their ability to respond to environmental stimuli, restructure themselves, and enable self-healing. When used in bioprinting—especially with the DLP method—these materials enhance the fabrication of dynamic, biologically relevant constructs [104,105]. Their value lies in mimicking the natural cellular microenvironment and promoting biological interactions [12]. For instance, shape memory polymers [13] can recover their form at threshold temperatures, making them ideal for muscle-mimicking or elastic tissue models. However, since most SMPs require thermal activation typically above physiological temperature (e.g., 37–45 °C), their use in biological environments may be limited without careful thermal control. Hydrogels, known for maintaining a moist environment, are widely utilized in soft tissue engineering. The incorporation of smart hydrogel systems with DLP printing has enabled more precise control over construct properties [106]. Moreover, stimuli-responsive materials such as pH- or heat-sensitive polymers contribute to the functionality and adaptability of printed constructs [107].

Owing to the micro-level resolution of DLP systems, smart materials can be aligned with intricate microarchitectures to fabricate constructs like vascular networks or neural interfaces. Such constructs adapt to environmental dynamics and support critical physiological functions. For example, shape memory polymers embedded in vessel-like channels enhance flexibility and performance in simulation environments [108,109]. Despite their promise, technical issues such as limited mechanical strength or light-sensitivity challenges exist. However, advances in material science—particularly in crosslinked hydrogel systems—are addressing these limitations and enabling more resilient structures [110,111]. In summary, the integration of smart materials into DLP-based bioprinting continues to expand the possibilities in regenerative therapies and advanced construct development, maintaining their role as a key driver in biomedical innovation.

**Figure 3:**
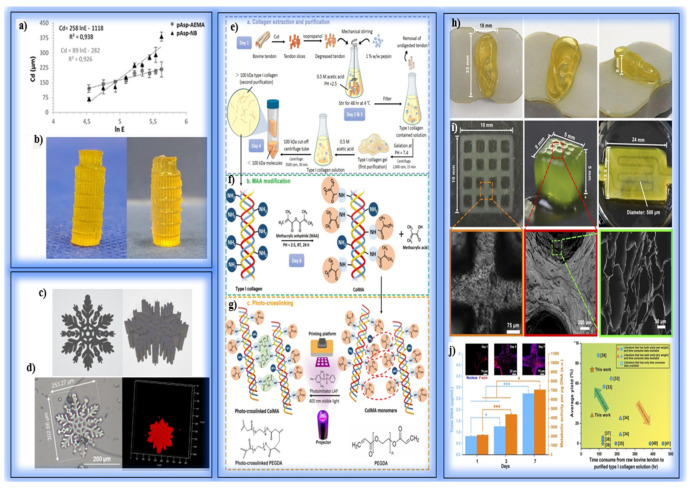
This figure presents various characterization results, including polymerization depth measurements, 3D-printed structures, collagen extraction and modification processes, and biocompatibility tests. (**a**) Working curves of DLP formulations exposed to 405 nm light at 23.16 mW/cm^2^. Cd represents cured resin thickness, and lnE is the natural logarithm of applied energy. (**b**) “Leaning Tower of Pisa” structures printed using PASP-AEMA (left) and PASP-NB (right) via DLP. (**c**) CAD design of a snowflake structure with dimensions of 200 µm (x) × 180 µm (y) × 100 µm (z). (**d**) Snowflake structures printed via 2PP using PBS solutions: (**b**) with 0.5 equiv. DTT and 1 mM P2CK, (**c**) with 1 mM P2CK alone. Optical microscopy (left) and confocal microscopy (right) images show swollen forms [112]. (**e**) Schematic of ColMA ink preparation: collagen extraction and purification. (**f**) MAA modification, resulting in methacrylate-functionalized collagen (ColMA). (**g**) Photo-crosslinking of ColMA under 405 nm light with LAP as a photoinitiator. (**h**) Various 3D-printed ColMA/PEGDA hydrogels: a large ear model (33 mm × 18 mm × 8 mm). (**i**) A small cubic model with vertical channels (5 mm × 5 mm × 5 mm), a microfluidic pad with 500 µm diameter channels (24 mm × 13.5 mm × 2 mm). Optical microscopy image of fibroblast proliferation on the scaffold on day 7 (scale bar = 75 µm), and SEM images showing the cross-sectional surface (scale bar = 200 µm) and porous structure (scale bar = 30 µm) of the hydrogel. (**j**) Statistical analysis of total DNA content and metabolic activity tests on days 1, 3, and 7. Confocal microscopy images show fibroblast morphology and distribution at corresponding time points (scale bar = 75 µm); comparison of collagen extraction time and yield from bovine tendons, indicating that this study (orange symbols) achieves higher yields in shorter times compared to previous studies [113].

De Grave et al. investigated the photo-crosslinkable structures of poly (aspartic acid) (pAsp) derivatives. The study evaluated the mechanical properties and cellular biocompatibility of hydrogel structures that can be processed by DLP and two-photon polymerization (2PP) methods. pAsp has great potential in biomedical applications as a biocompatible and biodegradable poly (amino acid). pAsp was modified with norbornene (NB) and aminoethyl methacrylate (AEMA) functional groups. The NB group was crosslinked using the thiol-ene step growth mechanism, while the AEMA group was crosslinked using the methacrylate-based chain growth mechanism. The hydrogels were crosslinked under UV-A light, and their mechanical properties were evaluated by photo-rheology measurements. The hydrogels crosslinked with NB reached the gelation point in 1.5 s, while the AEMA group gelated in 2.5 s. Crosslinking occurred more slowly due to oxygen inhibition in the chain growth mechanism. pAsp-AEMA hydrogels exhibited a stiffer structure with a storage modulus of 135.1 ± 4.7 kPa. pAsp-NB hydrogels showed lower stiffness with a storage modulus of 29.4 ± 1.3 kPa. The swelling ratio of the hydrogels was measured to be 7.4 ± 0.2 g/g for pAsp-AEMA and 45.7 ± 2.0 g/g for pAsp-NB, respectively. In cell culture experiments performed using the Alamar Blue test, it was observed that the hydrogels did not cause cellular toxicity. No significant decrease was observed in the metabolic activity of HEK293 cells surrounded by pAsp-NB and pAsp-AEMA hydrogels (Figure 3a). In DLP tests, pAsp-NB resins polymerized with lower energy (24 mJ/cm^2^), while pAsp-AEMA required 76 mJ/cm^2^. In 2PP tests, 45 mW laser power was required for pAsp-NB and 140 mW for pAsp-AEMA. Complex 3D structures, such as the “Leaning Tower of Pisa” design and the “snowflake” model, were successfully produced (Figure 3b–d). The study showed that different crosslinking mechanisms significantly affected the mechanical strength and swelling behavior of pAsp derivatives. pAsp-NB provided a homogeneous structure and rapid crosslinking, while pAsp-AEMA provided higher stiffness. These hydrogels are promising for personalized implants and tissue engineering applications in the biomedical field [112]. Han et al. developed a new ink by extracting type I collagen and combining its methacrylate derivative (ColMA) with PEGDA to produce biocompatible hydrogels for DLP printers (Figure 3e). In this study, the potential of collagen-based ink in bioengineering applications was investigated. In particular, ColMA/PEGDA ink stands out with its high resolution (50 µm) and mechanical strength properties. Collagen was extracted with acid solution and pepsin using bovine tendon and purified from impurities. Purified collagen was cleaned from protein and peptide residues using a 100 kDa filtration unit. In total, 0.6 wt% collagen solution was reacted with methacrylic anhydride (MAA) (Figure 3f). This process was carried out for 24 h at pH ≤ 2.5, and an average methacrylation rate of 88.3% was obtained. A 12 mg/mL ColMA solution was mixed with PEGDA (0.3 wt%), and 5% *w*/*v* LAP was used as a photoinitiator. A yellow-colored natural photoinhibitor was added to prevent excessive crosslinking. ColMA/PEGDA hydrogels showed stable viscosity at low temperatures (4–32 °C). Gelation was completed with 10 s of 405 nm light exposure (Figure 3g). The compressive modulus of the samples containing 0.6% ColMA was measured to be 137.8 ± 19.0 kPa. The hydrogels reached a swelling ratio of 832% within 48 h. In the 28-day degradation tests, a weight loss of 35.6 ± 2.9% was observed. In the tests, 50 µm wide gaps were successfully printed, and ear models were printed (Figure 3h). The hydrogel structures showed microfluidic channels with a diameter of 500 µm and porous structures with pore sizes between 50 and 150 µm. In the tests with NIH/3T3 fibroblast cells, the cells adhered well to the surface of the scaffold and proliferated rapidly. On day 7, the cells were observed to exhibit dendritic and well-stretched morphologies (Figure 3i). The study revealed that ColMA/PEGDA ink has suitable viscosity and biomechanical properties for DLP printers (Figure 3j). The ink provides high-resolution printability for personalized structures. In the future, it is recommended to extend the biocompatibility tests with different cell types [113].

**Figure 4:**
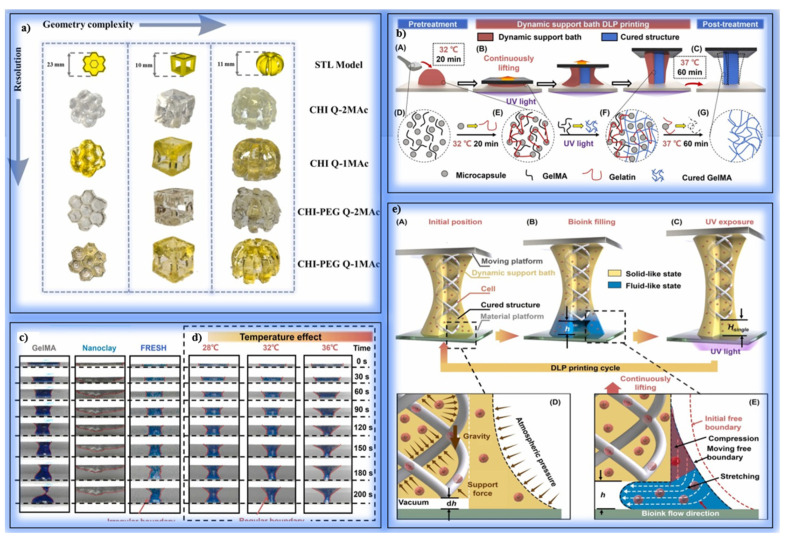
This figure illustrates various aspects of DLP-based 3D printing processes, the use of dynamic support baths (DSBs), and the characterization of printed structures. (**a**) Structures with varying geometry complexity and resolutions fabricated using different formulations (CHI Q-2MAc, CHI Q-1MAc, CHI-PEG Q-2MAc, and CHI-PEG Q-1MAc). STL models show the corresponding designs [114]. (**b**) Schematic of the DSB-DLP printing process: (**A**) pretreatment, (**B**) continuous lifting during UV light exposure, and (**C**) post-treatment at 37 °C. (**D**–**G**) Changes in the bioink composition throughout the process: (**D**) initial bioink with microcapsules, (**E**) after pretreatment, (**F**) after UV exposure, and (**G**) after post-treatment. (**c**) Optical images showing the shape of liquid bridges during DLP printing with different support baths: GelMA, nanoclay, and FRESH. (**d**) Temperature effects (28 °C, 32 °C, and 36 °C) on the DSB-DLP process, illustrating the boundary regularity of printed structures over time (0–200 s). (**e**) DLP printing cycle: (**A**) initial position of the dynamic support bath, (**B**) bioink filling the curing region, and (**C**) UV exposure. Insets (**D**,**E**) show the details of the support force, gravitational effects, and bioink flow characteristics during the printing cycle [55].

Zanon et al. used bio-derived photoactive dyes to fabricate chitosan methacrylate (CHI-MA) hydrogels with high resolution using the DLP method. Chitosan is an ideal polymer for tissue engineering due to its biocompatibility, biodegradability, and anti-inflammatory properties. However, the limited water solubility and low reactivity of chitosan make it difficult to fabricate complex 3D structures. HI-MA was mixed with PEGDA, and methacrylated quinizarine derivatives (Q-1MAc and Q-2MAc)—photoactive compounds derived from the anthraquinone dye quinizarine—were added at different ratios (Figure 4a). These dyes were methacrylated to enable photopolymerization under visible light and functioned as both crosslinking agents and photoreactive enhancers. A 3% *w*/*v* LAP was used as a photoinitiator. Light source: 405 nm LED. Layer thickness: 50 µm. Layer exposure time varied from 1.5 s to 8 s. Rheological tests measured the viscosity and crosslinking rates of the hydrogels. Mechanical strength was investigated using compression tests. Cell viability and proliferation were evaluated with C166-GFP endothelial cells. Formulations with Q-1MAc hardened in <1 s with rapid crosslinking. Hydrogels containing Q-2MAc showed higher storage modulus (G′) values due to the double methacrylate groups. Compression modulus was measured as 47 ± 2 kPa for Q-2Mac, and a 30% increase was observed compared to the reference hydrogels. Amplitude sweeps tests showed that structures containing Q-2MAc remained stable up to a 1000% strain rate. CHI-MA/PEGDA hydrogels reached a swelling ratio of 131%. The swelling ratio was around 165% in hydrogels supported with Q-1MAc and Q-2MAc. No cell death was observed in any hydrogels after 72 h of incubation. The cells spread on the surface and formed a monolayer. Quantitative DNA measurements showed that cell proliferation was high in hydrogels containing Q-2MAc. CHI-MA/PEGDA inks enriched with methacrylated quinizarin derivatives showed rapid curing and high-resolution printing performance in DLP printers. Mechanical strength was increased thanks to the crosslinking property of Q-2MAc. Hydrogels show promising results in terms of cell proliferation and biocompatibility and have been shown to have potential for tissue engineering applications [114]. Guoqiang Lu et al. examined the importance of photocuring 3D printing technologies in biomedical applications. Hydrogels are an ideal structural material, especially for tissue engineering and flexible electronic devices, thanks to their cell-friendly structures and water retention capacities. In this study, the printing techniques, materials, and application areas of hydrogels produced by photocuring are detailed. In their studies, they investigated the advantages, limitations, and application areas of DIW, SLA, DLP, CLIP, VAM, and TPP methods in detail. They determined that the inks used in the photocuring process generally consist of photocurable polymers, monomers, initiators, and additives. They observed that the initiator systems used differ according to the wavelength of light. As a result, they determined that hydrogels obtained with photocuring-based 3D printing technology offer personalized, high-resolution, and biocompatible structures for tissue engineering applications. However, they emphasized that difficulties such as low production speed and cost should be eliminated [115].

Soullard et al. investigated the printing of methacrylate carboxymethylcellulose (mCMC)-based hydrogel scaffolds for tissue engineering using additive manufacturing techniques such as DLP and TPP. Hydrogels are promising biomaterials for soft tissue repair due to their biocompatibility and mechanical adaptability. However, DLP has been found to be limited due to difficulties, such as the creation of hollow structures in the Z axis. For DLP, 2% mCMC with a methacrylation degree (DM) of 34% was used, along with 0.15% LAP as the photoinitiator. For TPP, 4% mCMC with a DM of 50% was used, along with 0.4% I2959 as the photoinitiator. DLP: 385 nm UV light, 18 mW/cm^2^ intensity. TPP: 532 nm femtosecond laser, 4 mW power. Rheological tests, swelling ratio measurements, and SEM analyses were performed. In DLP printing, the X and Y resolutions are limited to 85 µm, while the Z resolution is limited to 250–500 µm. The swelling ratio of hydrogels remained stable in PBS medium (SR ≈ 1.06), and no structural deterioration was observed. DLP is suitable for producing thin-walled microneedles, but successful results could not be obtained for hollow structures without the use of supporting photoabsorbers. In TPP printing, complex structures were successfully printed with a wall thickness of 10 µm. The hole diameters of the structures produced using TPP were reduced to 30 µm. This technique showed superior performance in soft tissue scaffold designs requiring micron-scale details. Although DLP is advantageous with its fast printing time and large surface area, there are resolution limitations in Z-direction printing. TPP is more effective in producing hydrogel structures with high resolution and complex geometry. In the future, more biocompatible photoabsorbers can be developed to increase the Z resolution of DLP. Furthermore, mCMC offers the potential for controlled drug release and cell growth in tissue engineering due to its water retention properties [116]. Li et al. developed an approach using a dynamic support bath (DSB) to enhance the structural stability of soft bioinks with low mechanical strength during photopolymerization bioprinting (Figure 4b). The incompatibility of conventional support baths with DLP arises from their static structures, which cannot meet the requirements of this method. The DSB provides support to the printed structure by dynamically moving in synchrony with the structure. A 5% (*w*/*v*) GelMA and 0.5% (*w*/*v*) photoinitiator (LAP) were used. DSB bioink enriched with microcapsules was prepared for high viscosity. A microcapsule suspension stabilized at pH 4.37 was used. The DLP device used emits UV light at a 365 nm wavelength. The bath temperature was kept constant at 32 °C, and the platform was moved 100 µm high for each layer (Figure 4c,d). The DSB remained constant throughout the structure with surface tension and atmospheric pressure differences. The Young Modulus of the DSB bioink was measured as 4.3 kPa after post-processing. While conventional GelMA bioinks have a strength of 2.6 kPa, DSB bioink provides a more stable structure. Thin-walled pipes, three-period minimal surfaces (TPMS), and branched catheters were successfully printed. The support bath protected the thin and complex structures from gravity and surface tension. HUVEC and hBMSC cells exhibited high cell viability in viability tests after 7 days of culture. YSF bioink promoted cell spreading and endothelial-like tissue formation. High expression of ZO-1 and occludin proteins was observed in the cells. Dynamic support bath (DSB)-DLP printing allows the use of bioinks with low mechanical strength to produce complex 3D structures (Figure 4e). In the future, improvements are suggested for loading biological contents (drugs or cells) using more microencapsulation methods. In addition, the use of biomaterials with lower stiffness for vessel-like structures can be investigated [55].

The versatile use of smart materials in DLP-based manufacturing processes offers new opportunities for functional organ and tissue fabrication, while the efficiency and accuracy of these processes also introduce several factors that can be further optimized. Key aspects, such as predicting material behavior during printing, minimizing errors in layering, and fine-tuning parameter settings to ensure biocompatibility, play a crucial role in producing highly precise and application-specific bio-constructs. At this point, AI- and ML-based technologies provide powerful tools for process optimization through data analysis and predictive modeling. The following section explores the integration of AI into DLP biomanufacturing and highlights the benefits this convergence brings to advanced biofabrication systems.

## 4. Integration of Artificial Intelligence and Machine Learning into DLP-Based Biomanufacturing Processes

AI and ML technologies are among the innovations that have revolutionized production processes in recent years and provide great advantages thanks to their integration into DLP-based biomanufacturing processes (Table 5). Biomanufacturing processes carried out using the DLP method are a technology that requires high precision in the creation of complex structures. In this context, artificial intelligence-supported systems contribute to the optimization of each step in the production process and improve critical parameters such as speed, cost, and quality control. ML algorithms play an important role in predicting the mechanical behavior of the photopolymer resins used in DLP production and determining appropriate production parameters. For example, the hardness, solubility, and biocompatibility values required in the production of a tissue model are simulated by AI algorithms, and the most appropriate parameters are obtained. This minimizes trial-and-error processes, shortens production time, and reduces material waste. In addition, deep learning algorithms can predict the behavior of biomaterials after printing and enable the modeling of cellular interactions.

AI-supported processes offer great potential, especially in personalized tissue and organ production. Personal biological characteristics obtained from patient data can be analyzed using AI algorithms, and tissue structures suitable for individual needs can be designed [15,16]. For example, in a heart valve model produced using the DLP method, an optimal design can be achieved by taking into account the patient’s tissue stiffness, elasticity, and biological structure [119]. This provides a great advantage in personalized medicine applications. Goh et al. developed an ML-based system for detecting anomalies in the 3D printing process. In the study, printing errors such as overextrusion and underextrusion were detected using the YOLOv3 and YOLOv4 deep learning models. The YOLO models detected errors at an early stage by analyzing images taken during the printing process and provided real-time feedback to the operator. The results showed that the YOLOv4 model had a higher accuracy rate compared to YOLOv3. The developed system made it possible to correct errors before they negatively affected print quality. This study shows that machine learning-based error detection systems can be used to improve the quality control process in additive manufacturing methods, such as DLP and FFF [120]. Fouly et al. evaluated the mechanical properties of 3D-printed samples of PLA-palm kernel composite materials using the Adaptive Neuro-Fuzzy Inference System (ANFIS) model. PLA blends containing different proportions of palm kernel particles were used in the printing. The experimental results showed that palm kernel addition above 15% caused a significant decrease in strength values. The ANFIS model showed high agreement with the experimental data in strength and hardness predictions at different material ratios. The study revealed that artificial intelligence-based models are an effective tool in the prediction of mechanical properties of biocomposite materials [121].

In this study, conducted by Rezapour Sarabi et al., an AI framework based on ML and deep learning (DL) was developed to predict the properties of microneedle arrays (MNAs) used in the production of biomedical devices. Microneedles provide advantages such as less painful drug applications and transdermal sample collection. The researchers produced 3D-printed microneedles using polylactic acid (PLA) using the FDM method and improved the geometric precision of the needles by applying chemical etching with a potassium hydroxide (KOH) solution. The properties of 2400 microneedle samples produced with different design parameters and etching times were evaluated using ML algorithms. DL models were trained to detect whether the produced microneedles were defective or not. CNN-based networks such as ResNet34, MobileNetV2, and ConvNeXt_Base were used for this purpose. In total, 240 samples were created with different geometric features (base diameter, height, angled shape) and etching parameters (molarity of solution and time), and 75% were allocated for training, 10% for validation, and 15% for testing. Support vector machines (SVMs) showed 86% accuracy, while the ConvNeXt_Base model showed the best performance, reaching 96% accuracy. As a result of the study, it was determined that long-term treatment with low molar concentrations (e.g., 3 M KOH) during the etching process was effective in eliminating design errors. The skin penetration and drug release capacities of the needles were tested. It was observed that the active ingredient-loaded microneedles were successfully applied to pig skin. This study demonstrated the effective use of AI to optimize the production process of 3D-printed microneedle arrays. In particular, deep learning-based quality control systems play a critical role in improving the performance of biomedical devices by identifying errors in microneedle production [122]. In addition, AI systems can automate quality control processes during production. Errors or deformations that may occur during production are detected in real time with machine learning-supported image processing techniques, thus making a flawless production process possible. This approach minimizes possible errors by increasing the mechanical durability and functionality of structures produced with bioprinters. However, the implementation of AI-based DLP bioproduction processes also brings with it some technical and ethical challenges. AI models that require training with large datasets can sometimes be limiting due to high computational power and time requirements. In addition, the accuracy and security of data are also important issues to consider. Especially when it comes to medical data, data privacy and ethical rules are of great importance. Chen et al. developed a biomimetic electronic skin sensor with object recognition and temperature sensing capabilities. The main purpose of their study was to design sensors with real skin-like sensing capabilities and to create a system that can be used in robotic and prosthetic applications. The device, designed using the DLP method, has two-mode sensors: one measures mechanical pressure, and the other measures temperature changes. The bionic skin sensor consists of thin platinum films placed on a flexible silicone base. Microstructures like surface texture were created in the sensor structure. The support vector machine (SVM) algorithm was used to classify the sensing data. In experiments conducted on objects with different material types and surface shapes, the sensor successfully identified objects with an accuracy rate of 98.11%. The sensor showed high flexibility and durability and gave accurate responses in different temperature ranges. The study showed that smart electronic skins can be used for object recognition and precise measurement in robotic systems. In the future, it is suggested that the sensor be developed for use in wearable electronic devices by increasing its lightness and energy efficiency [123].

Xiaohao Sun et al. investigated the potential of ML in 3D and 4D printing design. Their study aimed to model material behavior, optimize structure, and accelerate the design cycle in methods such as photopolymerization-based DLP. The predictive and feedback capabilities of ML models in the design process and their ability to improve deformation performance during printing were evaluated. Training was performed on large datasets obtained from material test data and 3D model simulations. Convolutional neural networks (CNNs), physics-informed neural networks (PINNs), support vector machines (SVMs), and generative adversarial networks (GANs) were used. As a result of the study, the PINN models showed significant accuracy in terms of mechanical deformation prediction and design feedback. GAN-based models showed promise in modeling complex geometry and material behavior. The study revealed that ML provides great contributions in optimizing design parameters for 3D/4D structures produced using the DLP method. However, challenges such as data diversity and model training times were also highlighted [124]. As a result, AI and ML offer major innovations in DLP-based biomanufacturing processes. These technologies not only increase production efficiency but also enable the implementation of individualized treatment approaches. In the future, it is anticipated that the capabilities of bioprinting technologies will be further expanded with more advanced AI models and data analysis methods. These developments indicate that AI-supported biomanufacturing will continue to play a critical role in areas such as regenerative medicine and organ repair [10,125].

## 5. Production of Functional Biological Structures with DLP Technology: Applications in Literature and Technical Challenges

Although functional biological construct manufacturing has made significant progress in recent years, it still faces several technical and ethical challenges. DLP-based biomanufacturing processes have some limitations in terms of the integration of biomaterials with complex structures and the production of functional tissues compatible with biological systems [30,126]. Overcoming technical challenges is critical to sustaining progress in this area. The mechanical strength and biocompatibility of biomaterials produced using the DLP method is one of the most fundamental technical limitations. When photopolymer materials are hardened by exposure to light, they can sometimes become brittle and lose their strength. This is a serious problem, especially in tissue structures that require flexibility [127]. In addition, the chemical structure of biomaterials is of great importance for increasing the vitality rate of tissues and long-term cell survival. The inability to provide vascularization in organ structures is one of the biggest technical obstacles encountered in creating large-scale tissue structures. Unless blood vessels and nerve networks are successfully integrated into organ models, the functionality of the produced tissues will be limited [128]. Another important technical challenge is the risk that the light source and curing process used in the DLP process may negatively affect cell viability. High energy density and long-term exposure to UV light can reduce cell viability. Therefore, the development of new-generation biocompatible photopolymer materials is important to increase the success of the process [129]. Despite all these difficulties, solutions to technical problems are being developed. In technical terms, new bioprinter modifications and biocompatible photopolymer materials are being produced that support the production of vascularized tissue structures using the DLP method. In light of these challenges and advancements, the following sections present selected case studies from the literature, categorized by material types and application areas, to illustrate how DLP technology is being adapted to overcome current limitations in functional biological structure fabrication.

### 5.1. Drug Delivery and Microinjection Systems

**Figure 5:**
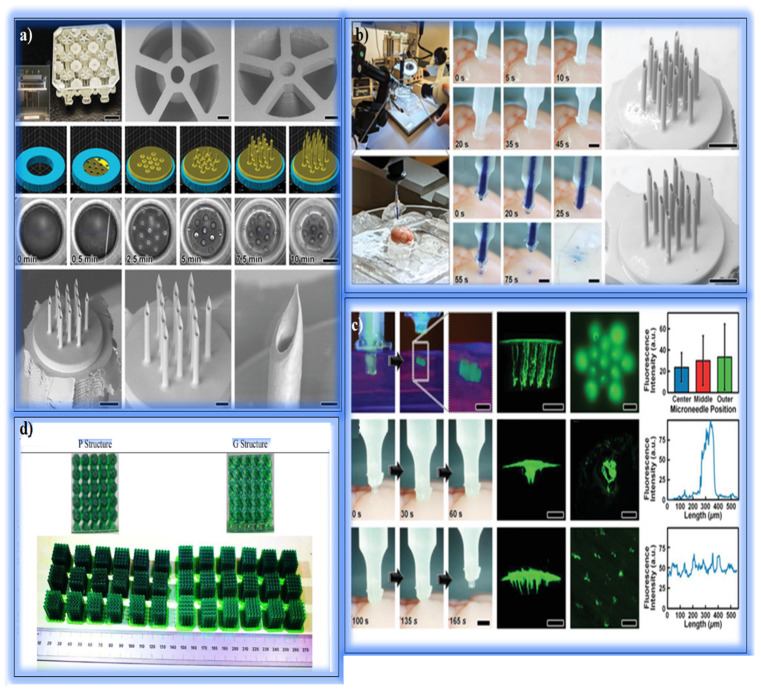
This figure presents various fabrication and characterization processes, including capillary array printing, microinjection experiments, fluorescence analysis, scaffold sintering, and structural comparison. (**a**) Capillary array structures printed via DLP and MNA printing via es-DLW: (top row) completed capillary arrays, CAD models, and SEM images of capillary structures with connecting bridges; (middle row) CAM simulations and real-time printing images at different time points (scale bar = 250 µm); (bottom row) SEM images of MNAs printed onto capillary structures, showing close-ups of microneedle tips (scale bars = 250 µm, 100 µm, and 25 µm, respectively). (**b**) Ex vivo MNA penetration and microinjection experiments: (left) experimental setup showing a custom-built nano-injector with an extracted mouse brain; (middle) sequential images showing MNA insertion and retraction (scale bar = 1 mm); (right) SEM images of MNAs after retraction (scale bar = 250 µm). (**c**) Fluorescence nanoparticle microinjections: (top row) in vitro microinjection images in 0.6% agar gels, including two-photon and wide-field fluorescence microscopy images (scale bars = 250 µm); (middle and bottom rows) ex vivo injection results showing fluorescence in mouse brain tissue, comparing conventional needles and MNAs (scale bars = 1 mm and 250 µm) [130]. (**d**) 3D-printed scaffolds with “P” and “G” structures fabricated using DLP technology, showing arrays of green scaffolds and top views of structural arrangements [133].

Sarker et al. developed high-precision hollow microneedle arrays (MNAs) using a hybrid DLP and direct laser writing (DLW) approach for targeted drug delivery and microinjection applications. By integrating DLP-fabricated microchannels with laser-written microneedle tips, the study achieved strong structural bonding and reliable fluid delivery. Ex vivo tests on brain tissue confirmed effective tissue penetration without leakage or mechanical damage, while pressure cycling demonstrated the durability of the system (Figure 5a–c). The design enabled a broader distribution of injected fluids compared to conventional needles, suggesting potential for applications in cellular therapies and neurological treatments. This hybrid manufacturing strategy offers a promising route for producing customizable microneedle systems with enhanced functional precision [130]. Bucciarelli et al. optimized DLP parameters for the fabrication of high aspect ratio (AR) microstructures used in microfluidic systems, such as organ-on-chip and lab-on-chip devices. By adjusting the UV exposure time and post geometry, the authors achieved improved mechanical stability and transparency in printed biocompatible resins. The optimal parameters supported the formation of structurally sound micro-pillars, enabling smooth PDMS mold replication and high-fidelity prototyping. This study highlights the advantages of DLP over traditional lithography in producing customizable, high-resolution microfluidic devices for biomedical applications [131]. Zhang et al. provided a comprehensive overview of DLP-based 3D printing applications in the medical field, emphasizing the advantages of DLP in producing complex, personalized structures with high resolution and speed. The technology has been successfully applied to anatomical modeling, dental prosthetics, microfluidic diagnostic devices, drug delivery platforms, and tissue scaffolds—especially those requiring vascularization. DLP’s compatibility with biocompatible and biodegradable materials enables its use in nerve repair, bone grafting, and organ simulation. Despite its promise, the method is still limited by its reliance on photopolymer-based materials, highlighting the need for broader material compatibility and further clinical validation. Overall, DLP is positioned as a key enabling technology for next-generation biomedical solutions [132]. As a result, addressing the technical and ethical challenges encountered in the process of producing functional organs and tissues is critical to ensuring the sustainability of biomedical research. In order to produce more durable, biologically functional, and ethical structures in the future, scientific studies and international collaborations need to be accelerated.

### 5.2. Soft Tissue and Skin Applications

**Figure 6:**
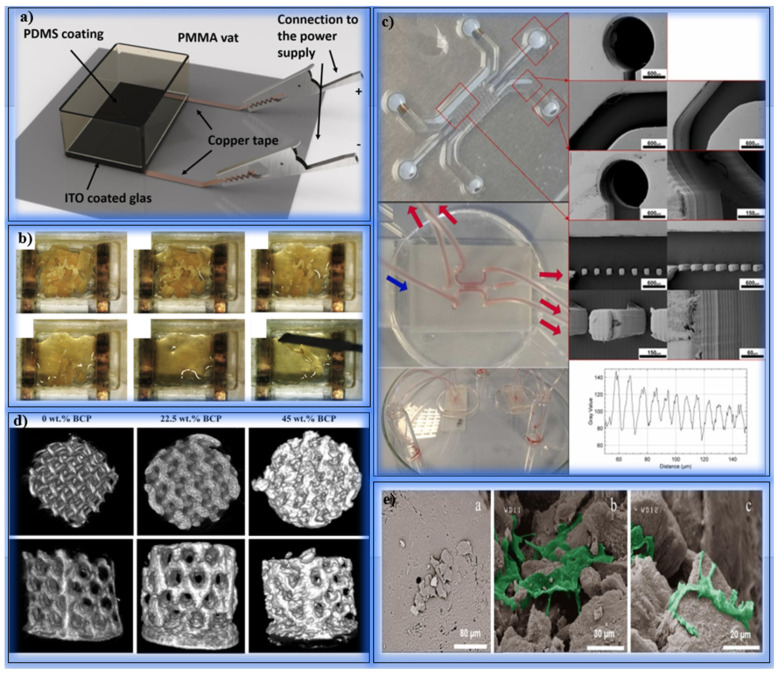
This figure illustrates the components, processes, and characterization results for the 3D printing system, including the PMMA vat setup, material melting stages, microfluidic chip analysis, scaffold reconstructions, and cell adhesion comparison. (**a**) Schematic of the heating stage comprising a PMMA vat with a PDMS coating seated on an ITO-coated glass. Copper tape provides a connection between the ITO coating and the power supply via alligator clips. (**b**) Time series of a heating vat filled and melted with caprolactone-co-tetramethylene carbonate (PCTAc) 75:25. Sequential images capture the melting process (frames from Supplementary Information Movie S1) [135]. (**c**) SEM images of microfluidic chip features: outlet, curved channel, and columns at different magnifications and angles (0° and 20° relative to the beam). The features exhibit well-defined structures in good agreement with the theoretical dimensions. A plot showing layer thicknesses between 20 µm and 25 µm. Microfluidic test showing one inlet (blue arrow) and multiple outlets (red arrows) [131]. (**d**) Micro-CT reconstructions of scaffolds with 0 wt.%, 22.5 wt.%, and 45 wt.% BCP content. Cross-sectional and lateral views show the internal structures of the scaffolds. (**e**) SEM images at 1000× magnification illustrating cell adhesion on scaffolds with (**a**) 0 wt.% BCP, (**b**) 22.5 wt.% BCP, and (**c**) 45 wt.% BCP [138].

Wang et al. synthesized biodegradable poly(ester) urethane acrylate (PEUAc) resins with varying molecular weights and evaluated their potential for fabricating soft tissue scaffolds using DLP technology. The study demonstrated that PEUAc resins with molecular weights of 4K and above achieved optimal performance, offering a balance between elasticity, processability, and biocompatibility. These formulations supported enhanced cell spreading and metabolic activity in fibroblast cultures and did not induce inflammatory responses in vivo. The mechanical properties of the scaffolds, particularly their low-modulus elasticity, made them suitable for mimicking elastomeric tissues such as skin and cartilage. This work highlights PEUAc as a promising resin system for developing personalized, biocompatible implants via DLP printing [134]. Kuhnt et al. developed a low-cost and easy-to-integrate heating platform to solve the challenges encountered in the production of high-viscosity biodegradable resins using DLP (Figure 6a). Resins used for the production of complex structures using DLP should generally have low viscosity (<10 Pa s). However, this requirement often requires the addition of solvent or reactive diluent (RD), which leads to a deterioration in mechanical properties and dimensional accuracy. In the study, a heating platform was designed using an indium-tin-oxide (ITO)-coated glass sheet and integrated into the DLP device. The heating device was connected to the power supply with copper strips, and the temperature control was provided between 30 °C and 120 °C. The heat distribution was evaluated using COMSOL simulation, and the rheological properties of different resin formulations were investigated. The resins were prepared with 30% RD using poly PCTAc- and PEUAc-based acrylates. The viscosity changes and curing behaviors of the resins against temperature increases were evaluated with rheological measurements during the heating phase. COMSOL simulations showed that bottom heating provided homogeneous temperature distribution over the entire surface of the resin bath. Side wall heating caused temperature differences in the center and prevented homogeneous curing. At 45 °C, the PCTAc 75:25 resin became suitable for printing by falling below the viscosity threshold value of 10 Pa s (Figure 6b). The PCTAc 90:10 resin, which was solid at 20 °C, gained a fluid structure at 45 °C, but structural integrity was lost when the RD ratio exceeded 50%. When the heating platform was used, complex 3D structures (e.g., porous biomaterials) were successfully produced, and no delamination was observed between layers. In printing without heating, deterioration in structure stability occurred due to an increase in the RD amount. The ITO-coated glass-based heating platform is a low-cost solution that can be easily integrated into DLP devices. This system enables the development of new resin formulations that can be used in biomedical applications by making high-viscosity resins suitable for printing. This temperature-controlled design has a wide potential for use in both academic research and industrial applications [135].

### 5.3. Bone Tissue Engineering

Guo et al. optimized DLP-fabricated hydroxyapatite (HA) scaffolds with bredigite (BR) doping using TPMS-based geometries to improve both mechanical and biological performance. By partially substituting Ca^2+^ with Mg^2+^ and Si^4+^ ions, BR doping enhanced microstructural integrity and promoted the formation of bioactive phases. Among various doping ratios, 10% BR content yielded the highest compressive strength (~33 MPa) and supported superior biomineralization. Increased BR levels also stimulated osteoblast proliferation and alkaline phosphatase (ALP) activity, indicating improved osteoconductivity. However, excessive doping (e.g., 20%) led to reduced density and mechanical strength due to increased microporosity. This study underscores the potential of BR-modified ceramic scaffolds in bone tissue engineering, particularly for enhancing osteogenic response while maintaining structural integrity [136]. Keshavarzan et al. explored the mechanical performance and failure mechanisms of triply periodic minimal surface (TPMS) structures fabricated by DLP for tissue engineering applications. Two TPMS geometries—primitive (P) and gyroid (G)—were produced with varying porosity levels (30–70%) to assess their load-bearing behavior under compression. The results showed that lower porosity (e.g., 30%) enhanced load capacity, while higher porosity improved deformation tolerance but increased susceptibility to shear band formation. The P-type structures exhibited better fatigue life and flexural behavior, whereas the G-type structures were more resistant to bending-induced damage. This study highlights the critical role of porosity and geometry in optimizing scaffold designs for mechanical reliability in regenerative applications [133].

Martinez et al. demonstrated the fabrication of high-resolution hydroxyapatite (HA) structures via DLP for bone tissue engineering. The study confirmed that sintered HA scaffolds provided favorable surface characteristics—such as moderate roughness and hydrophilicity—that supported osteoblast adhesion, proliferation, and early-stage osteogenic differentiation. Coating surfaces with biomolecules like Matrigel further enhanced cell adhesion. While the HA structures showed good initial biological responses, the authors highlighted the need for further optimization of mechanical strength and pore architecture for load-bearing applications. This work supports the potential of DLP-fabricated HA scaffolds in replicating complex bone tissue environments [137]. Bagheri Saed et al. investigated PLLA scaffolds reinforced with biphasic calcium phosphate (BCP) using the DLP method to enhance their suitability for bone regeneration. Incorporating 22.5% BCP improved biodegradability and cell adhesion without significantly compromising structural integrity, whereas a higher BCP content (45%) reduced mechanical strength due to increased ceramic loading. Micro-CT and SEM images (Figure 6d,e) confirmed homogeneous particle distribution and decreasing porosity with increasing BCP content. The 22.5% BCP scaffold exhibited the best overall balance in terms of mechanical properties, surface hydrophilicity, and cytocompatibility, highlighting its potential as a composite biomaterial for load-sensitive bone tissue applications [138].

## 6. Conclusions

This review provides a comprehensive synthesis of the current literature on the fabrication of functional biomedical components via DLP using smart materials. The analysis highlights that DLP technology, due to its high spatial resolution, sensitivity, and adaptability, holds considerable promise for biomedical applications. The ability to tailor printing parameters and material formulations enables DLP-based bioprinters to create patient-specific constructs. Technological discussions revealed that critical parameters—such as light source characteristics, photopolymerization mechanisms, and exposure settings—have a direct influence on the structural integrity and biocompatibility of printed scaffolds.

From a material science perspective, the incorporation of smart materials has demonstrated substantial benefits, including responsiveness to environmental stimuli, enhanced mechanical resilience, and self-healing capabilities. Additionally, recent advances in AI and ML have significantly improved the design, modeling, and process optimization of DLP-based biomanufacturing. ML algorithms, in particular, offer accurate predictions for material performance, process errors, and optimal fabrication parameters. Deep learning techniques further support the generation of complex tissue architectures and simulate cellular behaviors during fabrication.

Comparative assessments indicate that photopolymer-based hydrogels, bioceramics, and nanocomposites each offer distinct advantages for tissue engineering. Nonetheless, ongoing investigations are focusing on how material composition (e.g., composite formulations) and crosslinking mechanisms (e.g., photo-initiated vs. ionic or enzymatic) influence key outcomes, such as cellular viability, biodegradation dynamics, and mechanical robustness.

Overall, DLP-based techniques show strong potential for generating biodegradable microstructured scaffolds, although challenges persist—especially in large-scale production—such as structural deformation, interlayer defects, and limited mechanical strength. Addressing these issues will require the development of next-generation photopolymers with improved thermal and optical stability, as well as refined control over curing kinetics. The continued integration of smart materials and intelligent optimization systems is anticipated to enable the production of functionally graded, biologically adaptive, and clinically relevant constructs. Therefore, future research should prioritize scalable manufacturing strategies, enhanced material–cell interactions, and regulatory pathways for translational use in regenerative medicine and implantable device fabrication.

## 7. Future Perspectives

The integration of smart materials and DLP-based bioprinting holds immense promise for advancing biomedical engineering. However, several technical and translational challenges must be addressed to enable broader application and clinical implementation. First, the development of mechanically robust, biocompatible, and degradable materials remains a priority—especially those capable of responding to environmental stimuli, such as temperature, pH, or mechanical stress.

In terms of process scalability, DLP systems still face limitations in build volume, curing depth uniformity, and deformation during large-scale fabrication. Future improvements should focus on optimizing light source configurations, enhancing photoinitiator stability, and designing systems capable of multi-material printing with high fidelity. The integration of AI and ML into DLP workflows has the potential to significantly reduce error rates and improve printing accuracy. Deep learning models that simulate cell–material interactions, proliferation patterns, and post-fabrication behavior could accelerate the design of application-specific constructs. However, more work is needed to create standardized, validated datasets for training these models in biologically relevant contexts.

Another promising direction involves the fabrication of complex multilayered structures by combining materials such as bioceramics and photopolymer hydrogels. Such hybrid constructs can better mimic the hierarchical architecture of native tissues like blood vessels, cartilage, and skin. In addition, clinical validation remains a key step toward translational success. Future efforts should focus on testing DLP-produced scaffolds in real-world surgical simulations, as well as evaluating long-term biocompatibility and biofunctionality in vivo. Furthermore, the environmental impact of photo-curable resins and associated waste materials must be assessed through sustainability-oriented life cycle analyses.

Finally, increasing accessibility through the development of cost-effective, scalable, and eco-friendly bioinks will play a crucial role in expanding the reach of tissue engineering technologies across both research and clinical settings.

## Figures and Tables

**Table 2:** Comparison of DLP and other 3D bioprinting technologies.

Printing Technology	Resolution	Printing Speed	Cell Viability	Bioink Compatibility	Structural Complexity	Application Area
DLP	High (micron-level)	High (layer-wide projection)	Medium–high (in light-exposed environments)	Photopolymer-based bioinks	High (complex 3D structures possible)	Soft tissues, microstructures, vascular constructs
Extrusion-based	Low–Medium	Low–Medium	High (low mechanical stress)	Wide variety of bioinks	Limited (collapse risk)	Cartilage, bone, skin tissue
Inkjet-based	Medium–High	High	Medium (thermal/mechanical stress may occur)	Low-viscosity inks only	Limited (due to low viscosity)	Skin, neural tissue
Laser-assisted	High	Medium	High	Photoreactive bioinks	High	Vascular, skin, neural tissues

**Table 3:** Advantages, disadvantages, and application areas according to the materials used in the DLP method [77,78,79].

Type	Examples	Advantages	Disadvantages	Applications
Photopolymer Resins	Polyethylene glycol diacrylate (PEGDA) Polymethyl methacrylate (PMMA) Polycaprolactone (PCL) Epoxy acrylates	High resolution Biocompatible Easy to shape	Low mechanical strength UV light sensitive	Microvascular structures Biosensor models Dental applications
Smart Photopolymers	Shape memory polymers (SMP), Thermoresponsive hydrogels	Change in shape with heat, pH or light Biocompatible structure Long-term stability	Complex manufacturing protocol Material development costs	Cartilage replacements Soft tissue repairs Drug delivery devices
Hydrogels	Gelatin methacrylate (GelMA) Hyaluronic acid methacrylate (HAMA) Poly (ethylene glycol) (PEG) derivatives	Cell-friendly structure Soft tissue-like mechanical properties Customizability	Low hardness and strength Long curing times may be required	Bioprinting tissues Tissue repair models Blood vessel-like structure
Ceramic Modified Resins	Hydroxyapatite (HA) filled acrylates, Bioactive glass particles	Biocompatibility High hardness and strength Bone-like structure support	Brittleness due to high hardness Risk of non-homogeneous distribution	Bone tissue replacements Dental prosthetics Artificial joints
Nanocomposite Resins	Carbon nanotube-based resins Graphene oxide-added polymers	High surface area Electrical conductivity Reinforced mechanical strength	Complex production parameters Homogeneous mixing of additives is difficult	Microelectronic implants Smart biomedical devices

**Table 5:** Advantages of the integration of AI and ML over the DLP method [12,117,118].

Advantages	Details
Optimization of the Production Process	Production parameters (light exposure time, layer thickness, curing time) can be optimized with AI algorithms.
Error Detection and Correction	ML models can instantly detect errors (layer shifting, deformation) that may occur during production and suggest corrective steps.
Material Selection and Adaptation	With data analysis, appropriate options can be offered according to the mechanical strength, biocompatibility, and biodegradation behavior of different smart materials.
Predictive Performance Models	Before the production process, the mechanical and biological performances of the structures can be predicted by simulations.
Personalized Building Design	AI-powered design tools help design patient-based bio-constructs faster and more efficiently.
Data-Based Continuous Improvement	Data obtained from each production process are analyzed using ML algorithms, ensuring continuous improvement of processes.
Increased Speed and Efficiency	Thanks to optimum parameters, production time is shortened and energy/material waste is reduced.
Model Compatibility and Scalability	It can be easily adapted to production scale and new materials, increasing process flexibility.

## Data Availability

No new data were created or analyzed in this study. Data sharing is not applicable to this article.
